# An Improved NSGA-II Algorithm for Multi-Objective Optimization of Irregular Polygon Patch Antennas

**DOI:** 10.3390/mi16070786

**Published:** 2025-06-30

**Authors:** Zhenyang Ma, Jiahao Liu

**Affiliations:** 1 Institute of Science and Technology Innovation, Civil Aviation University of China, Tianjin 300300, China; zyma@cauc.edu.cn; 2 Key Laboratory of Civil Aircraft Airworthiness Technology, Civil Aviation University of China, Tianjin 300300, China; 3 College of Safety Science and Engineering, Civil Aviation University of China, Tianjin 300300, China

**Keywords:** improved NSGA-II, irregular polygonal patch antenna, multi-objective optimization, HFSS co-simulation

## Abstract

This paper presents an improved NSGA-II algorithm for the multi-objective optimization of irregular polygon patch antennas (IPPAs), improving convergence efficiency and Pareto front quality. The algorithm integrates adaptive mechanisms that dynamically adjust crossover and mutation rates based on generational progression, accelerating convergence while preserving solution diversity. Furthermore, a simulated annealing-inspired acceptance criterion is embedded during offspring generation to mitigate local optima trapping and enhance evolutionary robustness. A dual-objective formulation simultaneously minimizes antenna volume and maximizes operational bandwidth within the X-band. Optimization is executed via HFSS co-simulation, with detailed electromagnetic models ensuring physical realizability and design fidelity. The optimized antenna achieves a compact volume of 2807.6 mm^3^ and an operational bandwidth of 2.7 GHz. Experimental validation of fabricated prototypes demonstrates agreement with simulations, confirming the accuracy and reliability of the proposed method. These results demonstrate the effectiveness of the improved NSGA-II algorithm in addressing complex multi-objective design challenges and underscore its potential in advanced broadband antenna applications.

## 1. Introduction

Polygonal patch antennas are extensively utilized in modern communication systems due to their inherent adaptability in structural design, ease of fabrication, and their ability to deliver high performance in broadband, high-gain, and compact applications. Their versatility has led to widespread deployment in areas such as wearable devices [1], metasurfaces [2], and resonators [3], where stringent requirements for broadband operation [4], high gain [5], and advanced filtering are pronounced. While regular polygon patch antennas have been thoroughly investigated and broadly applied, the irregular polygon patch antennas (IPPAs) remain relatively underexplored, although some authors have proposed methods for their optimization. In [6], a genetic algorithm-based approach for miniaturizing rectangular microstrip patches was proposed. In [7], a genetic algorithm was employed to enhance the directivity of microstrip patch antennas. The systematic, multi-objective optimization of IPPAs presents unique challenges and design opportunities. Compared to regular polygon antennas, IPPAs offer greater design freedom and a larger number of adjustable parameters, enabling more sophisticated tuning of electromagnetic performance. However, this enhanced flexibility entails a substantial increase in computational complexity due to the growth in the number of design variables. Consequently, conventional parameter sweep methods become impractical for addressing such high-dimensional problems, thereby necessitating the use of heuristic or metaheuristic algorithms that can efficiently explore complex multi-objective design spaces.

In microwave device design, metaheuristic algorithms have already demonstrated significant success in optimizing antenna arrays [8,9], filters [10,11,12], and various antennas [13,14,15]. For antenna array optimization, performance evaluation is often decoupled from simulations, allowing for rapid assessment based on analytical models once the element characteristics are established. This significantly reduces computational overhead. In contrast, the optimization of antennas, filters, and other passive microwave components predominantly relies on computationally intensive full-wave simulations, such as those employing the Finite Element Method (FEM), with performance evaluation accounting for over 90% of the total optimization time. Surrogate-based optimization (SBO) [16], despite its ability to reduce simulation time, often struggles with high-dimensional parameter spaces due to limited generalizability and accuracy gaps between surrogate predictions and real device performance. Commercial simulation-based algorithms also suffer from slow convergence, especially in high-dimensional, multi-objective settings. These limitations underscore the necessity for scalable, robust optimization algorithms capable of effectively handling complex constraints and multiple objectives. While conventional approaches, such as Newton methods or region-based searches [17,18], remain popular, they are typically limited to problems with a small number of optimization variables (generally less than 20), which is insufficient for advanced antenna design requirements.

The research problem addressed in this paper falls within the domain of constrained multi-objective optimization problems (CMOPs), which are typically characterized by multiple conflicting objectives subject to various constraints. For such challenging optimization scenarios, multi-objective evolutionary algorithms (MOEAs) have been extensively studied due to their strong optimization capabilities. Based on their fundamental evolutionary strategies, existing MOEAs can be systematically classified into three major categories: Pareto-based methods [19], which optimize all objectives simultaneously by selecting feasible individuals using the Pareto dominance principle. Indicator-based methods [20] employ quality metrics to guide the evolutionary process. Decomposition-based methods [21] address multi-objective problems by decomposing them into several single-objective subproblems.

Among these approaches, the Non-dominated Sorting Genetic Algorithm II (NSGA-II) has been established as a widely adopted method owing to its operational simplicity, computational efficiency, and minimal parameter requirements. Nevertheless, NSGA-II demonstrates several inherent limitations: it tends to converge prematurely to local optima, especially when dealing with high-dimensional or severely constrained problems, and frequently experiences a gradual deterioration of population diversity during the evolutionary process. These deficiencies can significantly compromise both the quality and distribution of the Pareto front, consequently reducing the algorithm’s robustness and optimization effectiveness for complex antenna design problems [22,23]. Conventional antenna design methodologies predominantly rely on computationally intensive electromagnetic (EM) simulation-driven optimization, which becomes particularly prohibitive when dealing with high-dimensional design spaces [24]. The evaluation of numerous candidate solutions across expansive parameter domains, coupled with the need for precise multi-objective performance characterization, imposes substantial computational demands [25].

To overcome these limitations, this paper presents a framework that integrates evolutionary optimization with full-wave simulation tools. Specifically, we embed the improved NSGA-II algorithm within a co-simulation environment powered by the High-Frequency Structural Simulator (HFSS) [26,27]. The improved NSGA-II algorithm retains the strengths of the original framework, while featuring a dynamically adaptive mechanism that adjusts crossover and mutation rates according to the evolutionary stage [28], and incorporates an acceptance criterion inspired by simulated annealing during offspring generation [29,30]. These improvements are specifically designed to accelerate convergence, maintain solution diversity, and enhance the quality and stability of the Pareto front, thereby overcoming the limitations of NSGA-II in complex, high-dimensional multi-objective optimization scenarios.

The optimization problem is formulated to simultaneously minimize antenna physical volume and maximize operational bandwidth [31,32]. To adequately address this complex design challenge, our approach introduces an extensive set of 83 variable parameters into the optimization process, enabling unprecedented flexibility in antenna geometry exploration. The target frequency range is strategically selected as the X-band (8–12 GHz), which holds critical importance for communication systems [33]. Experimental validation by way of prototype fabrication and measurement demonstrates excellent correlation between simulated and empirical results, thereby confirming both the reliability and practical effectiveness of our proposed intelligent optimization strategy. The rest of this paper is organized as follows: Section 2 discusses the antenna structure and problem formulation. Section 3 details the improved algorithmic framework and computational methods. Section 4 discusses simulation results and experimental validation. Section 5 concludes the study and outlines future research directions.

## 2. Antenna Structure and Problem Description

In this section, we describe the designed antenna structure and formulate the associated optimization problem, which is defined by several constraints and objective functions. To ensure clarity and consistency, all symbols used in the subsequent equations are defined and summarized in Table 1.

### 2.1. Antenna Structure

**Figure 1:**
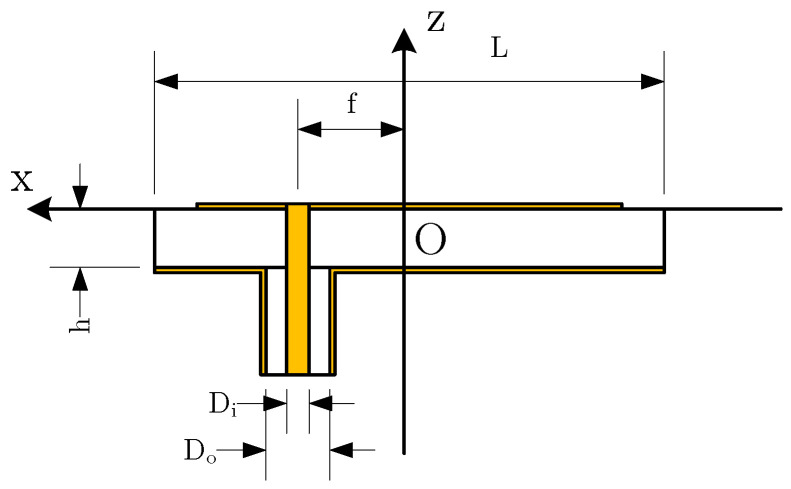
Side cut of antenna.

The polygon patch antenna designed in this study incorporates a dielectric substrate with a thickness of 1.6mm, chosen for its manufacturability and suitability for practical applications in advanced antenna designs. The antenna is excited through a coaxial feed line, as shown in Figure 1. This feed line is designed with a characteristic impedance of 50Ω, featuring an inner diameter ($D_{i}$) of 0.4343mm and an outer diameter ($D_{o}$) of 1mm. The dielectric medium inside the coaxial line is a vacuum, with a relative permittivity of 1.0. To minimize the impact of the exposed section of the feed line on the overall performance of the antenna, its length is fixed at 5mm in the simulation model. Figure 1 illustrates the sectional view of the antenna along the xOz plane. The upper and lower copper foils, which constitute the patch and ground plane, are modeled with an exaggerated thickness for clarity, although their actual thickness is 0.04mm.

**Figure 2:**
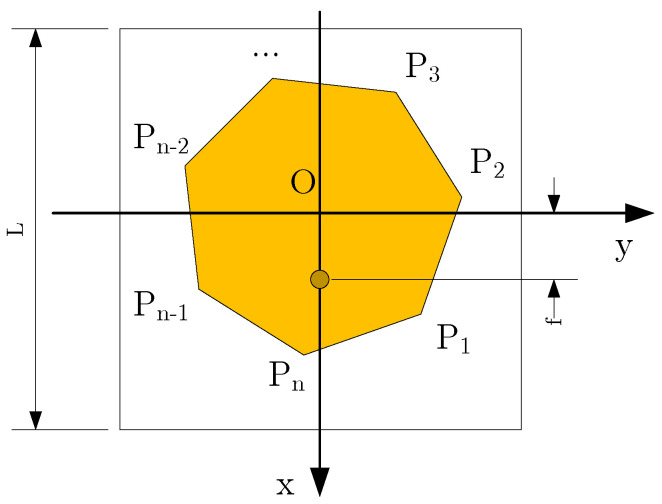
Vertical view of antenna.

The top view of the antenna is depicted in Figure 2, showing the polygonal patch structure placed on a square dielectric substrate. The center of the square substrate coincides with the origin *O*, and the position of the feed probe lies in the xOz plane. The maximum side length of the patch, denoted as *L*, and the base plate are designed as a fully conductive surface. The distance from the center of the feed probe to the *z*-axis is denoted as *f*. The polygon patch lies entirely within the xOy plane, with its vertices denoted in counterclockwise order as $P_{1},P_{2},\ldots ,P_{N}$, and their arguments are defined as shown in Equation (1). The coordinates of these vertices are expressed as $P_{1}\left(x_{1},y_{1}\right),P_{2}\left(x_{2},y_{2}\right),\ldots ,P_{N}\left(x_{n},y_{n}\right)$, and their corresponding angular positions are $\theta_{1},\theta_{2},\ldots ,\theta_{n}$.

(1) $$\theta =\left\{\begin{array}{cc} \arctan(\frac{y}{x}) & x>0\;\mathrm{and}\;y>0\\ \pi +\arctan(\frac{y}{x}) & x<0\\ 2\pi +\arctan(\frac{y}{x}) & x>0\;\mathrm{and}\;y<0\\ \frac{\pi }{2} & x=0\;\mathrm{and}\;y>0\\ -\frac{\pi }{2} & x=0\;\mathrm{and}\;y<0\\ 0 & x=0\;\mathrm{and}\;y=0 \end{array}\right.$$

### 2.2. Problem Description

To ensure the structural integrity of the antenna and to meet the practical manufacturing requirements, several geometric constraints are imposed on the polygon patch design:

1. Center of Gravity Constraint: The origin of the coordinate system must coincide with the polygon patch’s center of gravity to ensure that the generated polygon shape meets the requirements to avoid unnecessary deformation or overlap. This condition is expressed as:
  $$\begin{array}{c} \sum_{i=1}^{n}x_{i}=0\\ \sum_{i=1}^{n}y_{i}=0. \end{array}$$
2. Probe Distance Constraint: The distance *f* from the center of the feed probe to the origin must not exceed the average distance of the polygon vertices to the origin. This ensures that the feed probe remains within the bounds of the substrate and avoids physical interference:
  $$f\leq \frac{1}{n}\sum_{i=1}^{n}\sqrt{x_{i}^{2}+y_{i}^{2}}.$$
3. Vertex Connectivity Constraint: The edges of the polygon must connect vertices in the order of increasing angular positions to avoid self-intersections and maintain a valid polygon structure.
  $$\forall i\in \left[1,n-1\right],\theta_{i}\leq \theta_{i+1}.$$
4. Side Length Constraint: The largest side length of the polygon must be at least twice the average distance of its vertices from the origin to ensure the patch area fits within the dielectric substrate.
  $$\frac{L}{2}\geq \frac{1}{n}\sum_{i=1}^{n}\sqrt{x_{i}^{2}+y_{i}^{2}}.$$

The constraints described above outline the geometric and structural parameters essential for ensuring the physical feasibility, manufacturability, and performance reliability of the antenna design. These constraints establish the foundational guidelines for generating a valid polygonal patch structure that adheres to practical limitations. The primary optimization objectives of this study are defined to address the dual challenges of enhancing operational performance and achieving a compact design. Specifically, these objectives are as follows:

1. To maximize the bandwidth of the antenna within the frequency range of 8–12 GHz.
2. To minimize the physical volume of the antenna.

According to the variables defined above, the volume of the antenna can be expressed as:

(2) $$V=L^{2}h.$$

*V* represents the volume of the antenna, while the volumes of peripheral components, such as the coaxial line and SMA interface, are not considered in this calculation.

To calculate the bandwidth of the antenna, the first step is to derive the $S_{11}$ parameter curve, which describes the antenna’s impedance performance across a range of frequencies. In this study, the commercially available software ANSYS HFSS 2021 is utilized for this purpose. For clarity and abstraction, the calculation process can be expressed as a mapping function, $F:M\to F(R^{+},R^{-})$, where *F* maps the set of antenna models M to a corresponding relationship between positive real numbers $R^{+}$, representing the operating frequencies, and negative real numbers $R^{-}$, representing the $S_{11}$ parameter in decibels for passive antennas.

The antenna parameter curve, expressed as $S_{11}:\;R\to R$ and abbreviated as $S_{11}$, defines the bandwidth of the antenna as the frequency range where $S_{11}<-10\;\mathrm{dB}$. This bandwidth is calculated by identifying the turning points of the $S_{11}$ curve. However, since $S_{11}$ is computed from the mapping function *F*, it is represented as a discrete set of data points rather than a continuous function. Consequently, traditional gradient-dependent methods, such as the Newton method, cannot be used to determine the frequencies at which $S_{11}=-10\;\mathrm{dB}$. To address this challenge, a bisection-like method is employed, which is similar to the method used in numerical analysis to identify zero points. Specifically, if the frequency points $f_{n}$ and $f_{n+1}$ satisfy the condition in (3), $f_{n}$ is identified as a turning point.

(3) $$\left[S_{11}\left(f_{n}\right)+10\right]\left[S_{11}\left(f_{n+1}\right)+10\right]\leq 0.$$

The bandwidth calculation process is formalized in Algorithm 1. In this process, num denotes the total number of frequency points selected during frequency scanning. These frequency points, represented as $f_{i}$, are generally obtained at equal intervals through simulations or measurements conducted using a vector network analyzer. To plot the $S_{11}$ curve, the number of sampling points along the frequency axis is specified as num. The decision criterion defined in (3) is primarily valid for interior points within the sampling sequence. However, additional logic is required to handle edge points, ensuring that all relevant turning points are accurately identified, as demonstrated in Algorithm 1. The sequence *T*, constructed during the algorithm, is an ordered list of frequencies that satisfy $S_{11}<-10\;\mathrm{dB}$. Operations on this sequence include appending elements. For example, T∪a represents the operation of appending an element *a* to the end of the sequence. The length of the sequence *T* is calculated using the function length(). The indexing of the sequence begins from 0. For example, if the sequence *T* is given as:

T={a,b,d}

then:

$$\begin{array}{cc} & T\cup \{e\}=\{a,b,d,e\}\\ & T[0]=a\\ & T[1]=b \end{array}$$

**Algorithm 1:** Calculate bandwidth of antenna.calculateBandWidth$\left(S_{11}\right)$     T←{}     for i=0,1,…,num−1          if i=0  and  $S_{11}\left(f_{0}\right)<-10$               $T\leftarrow T\cup \{f_{0}\}$          if  i=num−1  and  $S_{11}\left(f_{i}\right)<-10$               $T\leftarrow T\cup \{f_{i}\}$          if  $\left[S_{11}\left(f_{i}\right)+10\right]\left[S_{11}\left(f_{i+1}\right)+10\right]\leq 0$               $T\leftarrow T\cup \{f_{i}\}$     m←length(T)     bw←0     for  i=0,1,…,m/2−1          bw←bw+T[2i+1]−T[2i]    **return** bw

The bandwidth bw corresponding to the frequency range is calculated as the sum of the differences between adjacent odd- and even-indexed elements in *T*. This ensures that each frequency range satisfying $S_{11}<-10\;\mathrm{dB}$ is accurately accounted for. Additionally, the frequency difference between two consecutive sampling points is given by (4), The absolute error in the calculated bandwidth bw can be expressed as ±2∆f.

(4) $$∆f=f_{i+1}-f_{i}\;\forall i\in \left[0,num-2\right].$$

The optimization objective is to maximize the total bandwidth of the antenna within the frequency range of 8–12 GHz. To express this as a minimization problem, the objective is reformulated as minimizing 12−bw. By integrating the constraints and objectives described above, the DOP/IPPA problem is formulated as shown in Equation (5).

(5) $$\begin{array}{cc} \; & \min\;\{V,12-bw\}\\ \; & \mathrm{subject}\;\mathrm{to}\\ \; & \left\{\begin{array}{cc} \sum_{i=1}^{n}x_{i}=0\\ \sum_{i=1}^{n}y_{i}=0\\ f\leq \frac{1}{n}\sum_{i=1}^{n}\sqrt{x_{i}^{2}+y_{i}^{2}}\\ \theta_{i}\leq \theta_{i+1} & \forall i\in [1,n-1]\\ h=1.6. \end{array}\right. \end{array}$$

## 3. Antenna Optimization

### 3.1. Principle of the Improved Nsga-II Algorithm

**Figure 3:**
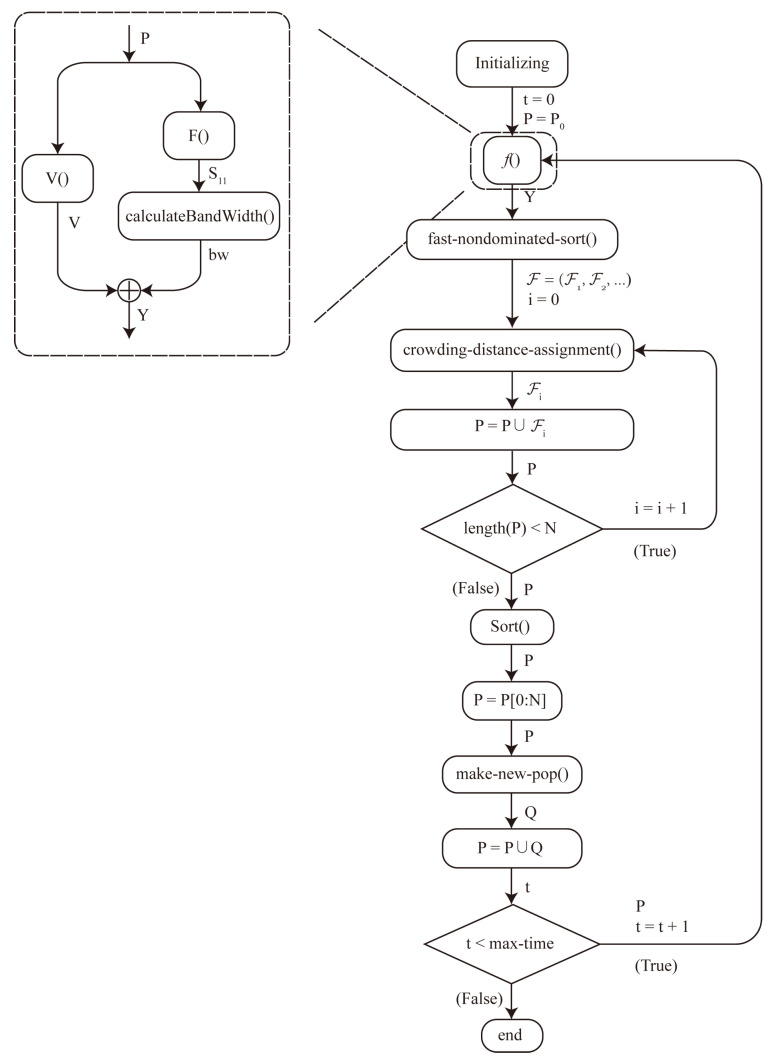
Flow diagram of the improved NSGA-II algorithm.

To address the dual-objective optimization of IPPAs, this study adopts an improved NSGA-II as the optimization framework. While the classical NSGA-II algorithm is effective in maintaining a diverse Pareto front and achieving convergence for multi-objective problems, it suffers from certain limitations, with the most significant being a tendency toward premature convergence and a loss of diversity, particularly in high-dimensional or constrained search spaces. To overcome these issues and further enhance optimization performance, this work introduces two major improvements: dynamic adaptation of genetic operator rates and a simulated annealing-inspired acceptance mechanism during offspring selection. As depicted in Figure 3, the improved NSGA-II operates as follows:

Step 1. Commence the algorithm by generating a two-dimensional random matrix *P*, which constitutes the initial population. In this matrix, the first dimension indexes distinct individuals *p*, and the second dimension encapsulates the various attributes $p^{i}$ of each individual.

Step 2. Evaluate the performance of each individual in the population by applying the objective function *f* to the population matrix *P*. This process generates the multi-dimensional objective function values, Y=f(P), which quantitatively evaluate the effectiveness of each individual in satisfying the optimization criteria.

Step 3. Execute non-dominated sorting on the population based on the objective values in *Y* to classify the individuals into multiple Pareto fronts. These fronts are represented as $F=(F_{1},F_{2},\ldots )$, where $F_{1}$ corresponds to the first Pareto front, containing the most optimal and non-dominated individuals, $F_{2}$ corresponds to the second front, and so forth.

Step 4. Compute the crowding distance for each individual within every Pareto front. This metric quantifies the relative proximity of neighboring solutions in the objective space, serving as a key indicator for preserving population diversity.

Step 5. The population is organised according to a hierarchical ranking system. Individuals are first ranked according to their Pareto front levels, with those in lower fronts indicating better fitness. Within each Pareto front, individuals are further ranked by their crowding distances, which measure the relative isolation of solutions in the objective space.

Step 6. Select *N* individuals from the sorted population to form the new population *P*, incorporating a simulated annealing-inspired acceptance mechanism.

Step 7. Apply genetic operators to the selected parent population *P* to generate a new population *Q*, with crossover and mutation rates dynamically adapted based on the evolutionary progress. Crossover combines genetic information from pairs of parents to produce offspring, while mutation introduces random variations to maintain genetic diversity. The new population is updated by combining the parent and offspring populations: P=P∪Q.

Step 8. Assess whether the current iteration count has surpassed the predefined maximum number of iterations. If this condition is satisfied, terminate the algorithm. If the condition is not met, the algorithm returns to Step 2 to continue the evolutionary process.

### 3.2. Antenna Representation and Data Structure

In the genetic algorithm, each individual in the population represents a unique antenna design, with its genes encoding specific geometric and structural parameters of the design. These parameters include the physical dimensions, the position of the feed point, and the coordinates of the vertices that define the *n*-sided polygon geometry of the antenna. The data structure used to represent an antenna as an *n*-sided polygon is formally defined as follows:

(6) $$p=[L,h,f,x_{0},y_{0},\ldots ,x_{n-1},y_{n-1}].$$

The parameters are defined as follows: *L* represents the length of the antenna, *h* denotes the thickness of the patch, *f* specifies the position of the feed probe, and $\left(x_{i},y_{i}\right)$ are the Cartesian coordinates of the *i*-th vertex of the polygon, ordered by their angular positions. Consequently, each individual *p* is a vector in $R^{2n+3}$, and the entire population *P* constitutes a matrix in $R^{N\times (2n+3)}$, where *N* is the population size.

Model *M* encompasses the antenna parameters and boundary conditions. In the implementation process, each individual *p* is mapped to *M* by directly incorporating its parameters without any additional mathematical transformations.Each *p* within the population *P* determines an $S_{11}$ curve. The function f() generates an array of $S_{11}$ curves, which are subsequently input into the function calculateBandWidth() to obtain the bandwidth information bw. This bandwidth information is represented as ${bw_{i}}_{N}$, where ${bw_{i}}_{N}\in R^{N}$.

After determining the bandwidth vector ${bw_{i}}_{N}$ and the volume vector ${v_{i}}_{N}$ for each individual in the population *P*, these vectors are concatenated to form a two-dimensional matrix $Y\in R^{N\times 2}$. Specifically, each row $Y_{i}$ of matrix *Y* comprises two elements: the volume $v_{i}$ and the bandwidth $bw_{i}$ corresponding to the *i*-th individual $p_{i}$ in the population. Subsequently, the population *P* is evaluated using the matrix *Y* as the basis for multi-objective optimization. The function fast_nondominated_sort() is applied to *Y* to perform non-dominated sorting, thereby identifying the Pareto front. This process ranks the individuals in *P* based on their performance across the two objectives—volume and bandwidth—without prioritizing one objective over the other. As a result, the non-dominated Pareto front comprises individuals that represent optimal trade-offs between volume and bandwidth. Following the identification of the Pareto front, the subsequent steps are carried out in accordance with the methodology presented in reference [34].

### 3.3. Initialization of the Antenna Population

The initialization process generates an initial population that complies with the problem constraints. Specifically, it ensures that antenna dimensions remain within the limits of the dielectric substrate and that the feed probe does not intersect the patch. The initialization procedure encompasses the following steps:

Step 1: Initiate the population initialization by generating *n* random radii ${\left\{r_{i}\right\}}_{i=0}^{n-1}$ from a uniform distribution $U(0,L_{\max})$ and *n* random angles ${\left\{\theta_{i}\right\}}_{i=0}^{n-1}$ from a uniform distribution U(0,2π), thereby establishing an initial distribution that encompasses the entire feasible design space for the polygonal antenna vertices. Transform each polar coordinate pair $\left(r_{i},\theta_{i}\right)$ into Cartesian coordinates $\left(x_{i},y_{i}\right)$ using the standard transformation equations, facilitating the accurate computation of the polygon’s centroid:

$$x_{i}=r_{i}\cos\left(\theta_{i}\right),\;y_{i}=r_{i}\sin\left(\theta_{i}\right).$$

Step 2: Determine the centroid $\left(\bar{x},\bar{y}\right)$ of the polygon by calculating the arithmetic mean of the Cartesian coordinates, serving as a pivotal reference point for subsequent spatial transformations:

$$\bar{x}=\frac{1}{n}\sum_{i=0}^{n-1}x_{i},\;\bar{y}=\frac{1}{n}\sum_{i=0}^{n-1}y_{i}.$$

Translate all vertices so that the centroid of the polygon aligns with the origin of the coordinate system by subtracting the centroid coordinates from each vertex, thereby centralizing the polygon for uniform scaling and rotation:

$$x_{i}\leftarrow x_{i}-\bar{x},\;y_{i}\leftarrow y_{i}-\bar{y}\;\forall i\in \left[0,n-1\right].$$

Step 3: Convert the translated Cartesian coordinates $\left(x_{i},y_{i}\right)$ back to polar coordinates $\left(r_{i},\theta_{i}\right)$ using the inverse transformation, preparing the data for angular sorting:

$$r_{i}=\sqrt{x_{i}^{2}+y_{i}^{2}},\;\theta_{i}=\arctan\left(\frac{y_{i}}{x_{i}}\right).$$

Sort the vertices in ascending order based on their angular coordinates $\theta_{i}$ using an efficient fast sorting algorithm to ensure that the consecutive connection of vertices forms a non-intersecting polygon, thereby preventing overlapping areas:

$$\theta_{0}\leq \theta_{1}\leq \ldots \leq \theta_{n-1}.$$

Step 4: Compute the average radius $\bar{r}$ from all adjusted vertices to the origin, which is instrumental in determining the feasible range for the antenna’s side length *L* and the probe position *f*:

$$\bar{r}=\frac{1}{n}\sum_{i=0}^{n-1}r_{i}.$$

Generate the side length *L* from a uniform distribution $U(\bar{r},L_{\max})$ to ensure that the antenna’s dimensions remain proportionate and within the desired range, contributing to optimal performance and manufacturability:

$$L∼U(\bar{r},L_{\max}).$$

Generate the probe position *f* from a uniform distribution $U(0,\bar{r})$, thereby maintaining the probe within the feasible design space and ensuring optimal antenna performance:

$$f∼U(0,\bar{r})$$

Step 5: Assemble the individual vector *p* by integrating the generated parameters *L*, *h*, *f*, and the sorted Cartesian coordinates $\left(x_{i},y_{i}\right)$ as defined in Equation (6), thereby creating a comprehensive representation of a unique antenna design:

$$p=[L,h,f,x_{0},y_{0},\ldots ,x_{n-1},y_{n-1}].$$

Step 6: Incorporate the generated individual vector *p* into the population *P*, and if the population size length(P)<N, repeat the entire process starting from Step 1; otherwise, conclude the initialization process, ensuring the creation of a population *P* containing *N* diverse and valid antenna designs.

### 3.4. Generation of New Individuals

The generation of new individuals within the NSGA-II-DOP/IPPA algorithm is pivotal for exploring the solution space and enhancing population diversity. This process is facilitated by two primary genetic operators: mutation and crossover. To optimize search efficiency and accelerate convergence, adaptive mechanisms are integrated into both mutation and crossover operations. Specifically, both the mutation rate and crossover rate decrease as the number of generations increases. For each generation *g*, the mutation rate $r_{v}\left(g\right)$ and crossover rate $r_{c}\left(g\right)$ are defined as:

$$r_{v}\left(g\right)=r_{v0}\left(1-\frac{g}{G_{\max}}\right),$$

$$r_{c}\left(g\right)=r_{c0}\left(1-\frac{g}{G_{\max}}\right),$$

where $r_{v0}$ and $r_{c0}$ are the initial mutation and crossover rates, and $G_{\max}$ is the maximum number of generations. This adaptive adjustment ensures that the algorithm favors exploration in the early stages and gradually shifts towards exploitation as it converges.

#### 3.4.1. Mutation Strategies for Generating Offspring

Mutation serves as a critical mechanism for introducing genetic diversity by randomly modifying an individual’s specific attributes. These attributes include the side length *L*, probe position *f*, and the vertices coordinates of the polygon. The mutation process is executed through the following steps:

Step 1: Randomly select an individual *p* from the current population *P* to undergo mutation, ensuring an equal opportunity for all individuals to contribute to population diversity.

Step 2: Generate a random number $r^{\left(0\right)}$ from a uniform distribution U(0,1). Based on this value, determine which attribute to mutate according to the following rules:

$$\left\{\begin{array}{cc} 0\leq r^{\left(0\right)}<r_{L} & \mathrm{Mutate}\;L\\ r_{L}\leq r^{\left(0\right)}<r_{L}+r_{f} & \mathrm{Mutate}\;f\\ r_{L}+r_{f}\leq r^{\left(0\right)}\leq 1 & \mathrm{Mutate}\;a\;\mathrm{vertex}, \end{array}\right.$$

where $r_{L}$ and $r_{f}$ represent the probabilities of mutating *L* and *f*, respectively, ensuring that $r_{L}+r_{f}\leq 1$. If none of the above conditions are met, a vertex is selected for mutation.

Step 3: If *L* is selected for mutation, generate a new side length $L^{*}$ from a uniform distribution $U(\bar{r},L_{\max})$, and replace the original *L* with $L^{*}$ in the individual *p* to explore new dimensions of the antenna design. Similarly, if *f* is selected for mutation, generate a new probe position $f^{*}$ from a uniform distribution $U(0,\bar{r})$, and replace the original *f* with $f^{*}$ in the individual *p* to optimize the probe placement within the feasible design space.

If a vertex is selected for mutation, perform the following sub-steps:

1. Selection of Vertex Index: Generate a random number $r^{\left(1\right)}$ from a uniform distribution U(0,1). The index *i* of the vertex to mutate is then determined as follows:
  $$i=\lfloor r^{\left(1\right)}\times n\rfloor .$$
2. Generation of New Vertex Coordinates: Generate new polar coordinates $\left(r_{i}^{*},\theta_{i}^{*}\right)$ from uniform distributions $U(0,L_{\max})$ and U(0,2π) respectively:
  $$r_{i}^{*}∼U\left(0,L_{\max}\right),\;\theta_{i}^{*}∼U\left(0,2\pi \right).$$
  Convert polar coordinates to Cartesian coordinates $\left(x_{i}^{*},y_{i}^{*}\right)$:
  $$x_{i}^{*}=r_{i}^{*}\cos\left(\theta_{i}^{*}\right),\;y_{i}^{*}=r_{i}^{*}\sin\left(\theta_{i}^{*}\right).$$
3. Updating Vertex Coordinates: Replace the original coordinates $\left(x_{i},y_{i}\right)$ with the new coordinates $\left(x_{i}^{*},y_{i}^{*}\right)$ in the individual *p*.
4. Recalculation of Centroid and Re-sorting: Recalculate the centroid of the updated polygon and translate the vertices to align the centroid with the origin, followed by angular sorting to maintain a valid polygon structure.
5. Adjustment of L and f: If the new side length *L* is less than $\bar{r}$, it is regenerated from the uniform distribution $U(\bar{r},L_{\max})$. Similarly, if the new probe position *f* exceeds $\bar{r}$, it is regenerated from $U(0,\bar{r})$. These adjustments ensure that all design parameters remain within feasible limits.

Step 4: Assemble the new individual *q* with the updated parameters and add it to the offspring population *Q*.

#### 3.4.2. Crossover Mechanisms for Generating Offspring

Crossover facilitates the combination of genetic information from two parent individuals to produce offspring, thereby promoting the exploration of new regions within the design space. To enhance the efficiency and adaptability of the crossover process, an adaptive crossover rate is employed, which decreases as the number of generations increases. The crossover process is executed through the following steps:

Step 1: Select two distinct individuals $p_{1}$ and $p_{2}$ from the current population *P* to serve as parents for crossover. Extract the vertex sequences of both parents:

$$E_{1}=\left\{x_{0}^{1},y_{0}^{1},\ldots ,x_{n-1}^{1},y_{n-1}^{1}\right\},$$

$$E_{2}=\left\{x_{0}^{2},y_{0}^{2},\ldots ,x_{n-1}^{2},y_{n-1}^{2}\right\}.$$

Step 2: Adaptively determine the crossover points based on the population’s diversity. Randomly select two distinct indices $i_{\mathrm{begin}}$ and $i_{\mathrm{end}}$ such that $0\leq i_{\mathrm{begin}}<i_{\mathrm{end}}<n$, allowing the algorithm to balance exploration and exploitation effectively. Exchange the vertex segments between the selected indices to produce new vertex sequences for the offspring:

$$\begin{array}{cc} E_{1}^{*}=\{ & x_{0}^{1},y_{0}^{1},\ldots ,x_{i_{\mathrm{begin}}}^{2},y_{i_{\mathrm{begin}}}^{2},\\ \; & \ldots ,x_{i_{\mathrm{end}}}^{2},y_{i_{\mathrm{end}}}^{2},\ldots ,x_{n-1}^{1},y_{n-1}^{1}\} \end{array}$$

$$\begin{array}{cc} E_{2}^{*}=\{ & x_{0}^{2},y_{0}^{2},\ldots ,x_{i_{\mathrm{begin}}}^{1},y_{i_{\mathrm{begin}}}^{1},\\ \; & \ldots ,x_{i_{\mathrm{end}}}^{1},y_{i_{\mathrm{end}}}^{1},\ldots ,x_{n-1}^{2},y_{n-1}^{2}\}. \end{array}$$

Step 3: For each offspring, recalculate the centroid, translate the vertices to align the centroid with the origin, and sort the vertices by their angles to ensure a valid polygon structure. Subsequently, regenerate *L* and *f* if they violate the constraints:

$$L_{i}^{*}=\left\{\begin{array}{cc} \mathrm{Regenerate}\;\mathrm{from}\;U({\bar{r}}_{i},L_{\max}) & \mathrm{if}\;L_{i}^{*}<{\bar{r}}_{i}\\ L_{i}^{*} & \mathrm{otherwise} \end{array}\right.$$

$$f_{i}^{*}=\left\{\begin{array}{cc} \mathrm{Regenerate}\;\mathrm{from}\;U(0,{\bar{r}}_{i}) & \mathrm{if}\;f_{i}^{*}>{\bar{r}}_{i}\\ f_{i}^{*} & \mathrm{otherwise}. \end{array}\right.$$

Step 4: Adaptively adjust the crossover rate $r_{c}\left(g\right)$ based on the population’s diversity and convergence status. Combine the revised parameters $L_{i}^{*}$ and $f_{i}^{*}$ with the updated vertex sequences $E_{1}^{*}$ and $E_{2}^{*}$, respectively, while retaining the fixed patch thickness *h*, to form the offspring individuals $q_{1}$ and $q_{2}$ as defined in Equation (6):

$$q_{j}=\left[L_{j}^{*},h,f_{j}^{*},x_{0}^{j},y_{0}^{j},\ldots ,x_{n-1}^{j},y_{n-1}^{j}\right]\;\mathrm{for}\;j=1,2.$$

Step 5: Add both $q_{1}$ and $q_{2}$ to the offspring population *Q*.

The integration of adaptive mutation and crossover mechanisms enables the algorithm to dynamically adjust its exploration and exploitation strategies in response to the changing characteristics of the population. This adaptability helps the algorithm escape local optima and accelerates convergence towards the Pareto-optimal front. Consequently, the overall performance of the antenna optimization process is significantly improved, leading to more efficient and effective optimization.

#### 3.4.3. Selection Procedures in Evolutionary Process

Achieving an optimal balance between exploration of the search space and exploitation of promising solutions is paramount in multi-objective optimization to secure a diverse and high-quality Pareto front. While the classical NSGA-II algorithm excels in efficiency, it exhibits inherent limitations such as susceptibility to premature convergence and stagnation in local optima, particularly when handling high-dimensional or constrained problems. To enhance the robustness of the search process and mitigate these deficiencies, we introduce a simulated annealing-inspired offspring acceptance mechanism. This approach embeds controlled stochasticity into the selection phase, enabling the algorithm to escape local optima during early generations while progressively refining convergence toward globally optimal solutions.

The proposed mechanism draws inspiration from simulated annealing, which strategically accepts inferior solutions with a decaying probability governed by a temperature parameter. Within the improved NSGA-II framework, this mechanism operates during offspring selection, determining whether newly generated individuals enter the subsequent population. Specifically, in each generation, if a newly generated offspring is dominated by its parent, it may still be accepted into the next population with a certain probability. This probability is determined by the difference in objective values between the offspring and its parent, as well as the current temperature parameter. As the evolution progresses, the temperature gradually decreases, thereby reducing the acceptance probability over time, in a manner analogous to the cooling schedule in simulated annealing. By probabilistically accepting dominated offspring, the algorithm amplifies its exploratory capabilities, thereby reducing the risk of premature convergence while preserving population diversity. The Selection process evaluates offspring against their parents based on multi-objective dominance criteria, as detailed below:

Dominance Assessment: For each offspring generated during the evolutionary process, its objective vector is evaluated against the corresponding vectors of its parent solutions. Offspring that exhibit dominance over their parents are unconditionally incorporated into the subsequent population. This approach ensures the retention of superior solutions, facilitating steady advancement toward the Pareto optimal front.

Non-Dominated Assessment: When an offspring and its parent solutions are mutually non-dominated, the selection process prioritizes the candidate with a greater crowding distance within the objective space.

Probabilistic Acceptance: If an offspring is dominated by its parent solution, it is classified as inferior under standard evaluation criteria. The algorithm employs a probabilistic acceptance strategy derived from the Boltzmann distribution, as utilized in simulated annealing. This method allows for the occasional acceptance of dominated offspring, with the probability determined by the relative difference in objective vector quality and a temperature parameter that decreases over iterations. Such an approach enables broader exploration of the solution space and mitigates the risk of convergence to suboptimal regions. The probability of acceptance can be expressed as:

$$P_{\mathrm{accept}}=\left\{\begin{array}{cc} 1 & \mathrm{if}\;∆F\leq 0\\ \exp\left(-\frac{∆F}{T}\right) & \mathrm{if}\;∆F>0, \end{array}\right.$$

where ∆F represents the difference in objective function values between the offspring and parent, *T* denotes the current temperature parameter.

The adaptive mechanism dynamically adjusts the crossover and mutation probabilities at the commencement of each generation. This adjustment is determined by a straightforward calculation involving the current generation number and the predefined maximum number of generations. The computational complexity of the NSGA-II algorithm is primarily dictated by its non-dominated sorting procedure. The adaptive scheme functions as an independent, preparatory step that does not interact with or alter this core sorting logic. Consequently, the computational complexity of the non-dominated sorting procedure remains unaffected. Therefore, the integration of the adaptive method does not increase the fundamental algorithmic complexity of the NSGA-II framework.

## 4. Simulation and Measurement

### 4.1. Parameter Settings

In the optimization of polygonal antenna designs, the selection of key parameters is essential to achieving an optimal balance between computational efficiency and solution quality. To ensure the reliability and reproducibility of our results, the key parameters for our simulation experiments were explicitly defined as follows. The antenna was modeled as an irregular polygon with n=40 sides, providing a high degree of geometric variability necessary for exploring a broad design space. The population size was set to N=82 individuals per generation, offering a substantial pool of potential solutions while maintaining manageable computational requirements. Genetic diversity within the initial population plays a pivotal role in the performance of evolutionary algorithms. To foster this diversity, both the initial mutation and crossover rates are set to 50%, introducing significant diversity during the early stages of optimization. The mutation and crossover rates are adaptively decreased over successive generations to guide the algorithm in transitioning from exploration to exploitation. The initial temperature is set to $T_{0}=1000$, enabling significant exploration in early generations, and the cooling rate is α=0.95. The maximum side length of the antenna was constrained to $L_{\max}=100\;\mathrm{mm}$ to ensure manufacturability and practical application. The probabilities for mutating the side length *L* and the probe position *f* were each assigned a value of $r_{L}=r_{f}=\frac{1}{6}$, ensuring a balanced distribution of genetic variations across different design parameters. By selecting and adjusting these parameters, the proposed optimization framework effectively balances exploration and exploitation, reduces computational complexity, and ensures the generation of practical and high-performance antenna designs.

### 4.2. Calculation Results

**Figure 4:**
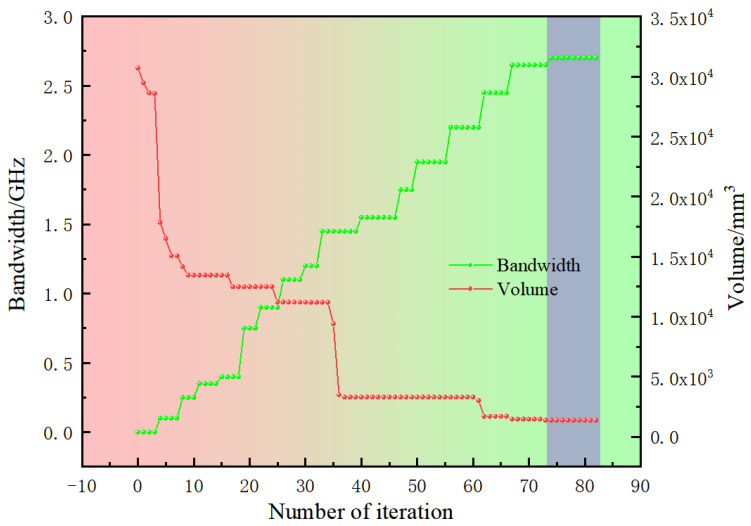
The optimization process for antenna volume and bandwidth.

Simulations were conducted using the parameter settings outlined and the adaptive optimization algorithm. The optimization process was carried out over 80 generations, evaluating a total of 6698 individual antenna designs. Each simulation required an average of two minutes per individual, leading to a cumulative computation time of approximately one week. The computations were performed on a high-performance computing system equipped with dual Intel Xeon Gold 6140 processors and 128 GB of RAM. Figure 4 depicts the optimization trajectory, emphasizing antenna volume and bandwidth as critical performance metrics. These metrics were employed to evaluate the convergence of the optimization algorithm. The optimization process was monitored across multiple generations, and the results indicate that significant improvements in the Pareto front ceased beyond the 70th generation, indicating the algorithm had effectively converged to optimal solutions by this stage.

**Figure 5:**
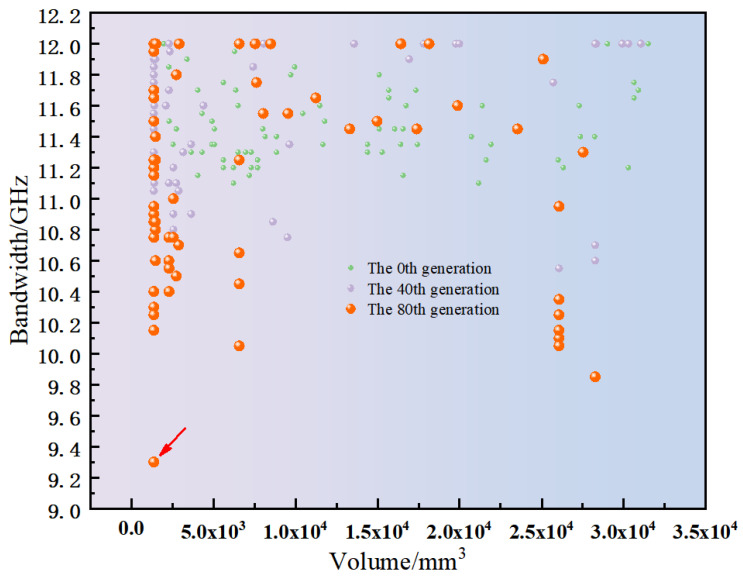
The calculation results for the 0th, 40th, and 80th generations.

Figure 5 illustrates the population distribution at the 0th, 40th, and 80th generations, with antenna volume plotted on the horizontal axis and 12−bandwidth on the vertical axis. The data demonstrate a consistent improvement in both antenna volume and bandwidth. Notably, substantial enhancements are observed between the initial (0th) and final (80th) generations, demonstrating the progressive optimization of solutions by the algorithm. The optimized antenna achieves a compact volume of $2807.6\;{\mathrm{mm}}^{3}$ and an enhanced bandwidth of 2.7 GHz. These results validate the algorithm’s effectiveness in simultaneously minimizing physical dimensions and maximizing operational bandwidth. An analysis of the convergence and stability of the multi-objective optimization process was conducted to substantiate the robustness of the proposed algorithm. The convergence behavior is primarily attributed to the dynamically adaptive crossover and mutation operators, which adjust their rates based on the population diversity and fitness progression. This mechanism ensures a broad exploration of the solution space in the initial generations, gradually shifting to intensive exploitation as the Pareto front matures. Furthermore, stability was evaluated through multiple independent executions. The resultant Pareto fronts demonstrated consistency, a characteristic enhanced by the simulated annealing acceptance mechanism. Consequently, the proposed method reliably identifies the Pareto front, underscoring its stability and repeatability.

**Figure 6:**
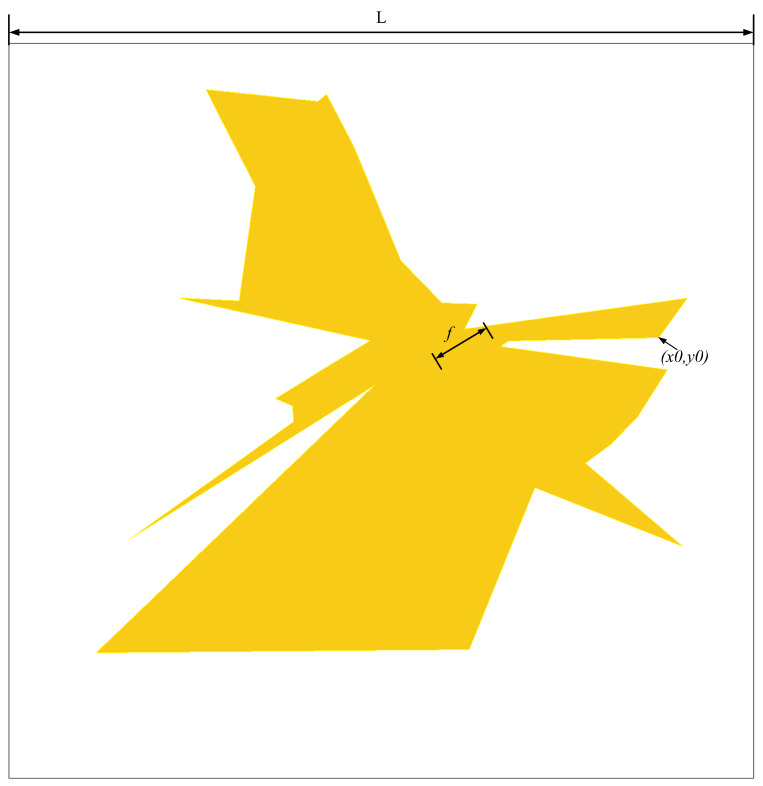
Schematic diagram of the upper patch of the broadband antenna.

**Figure 7:**
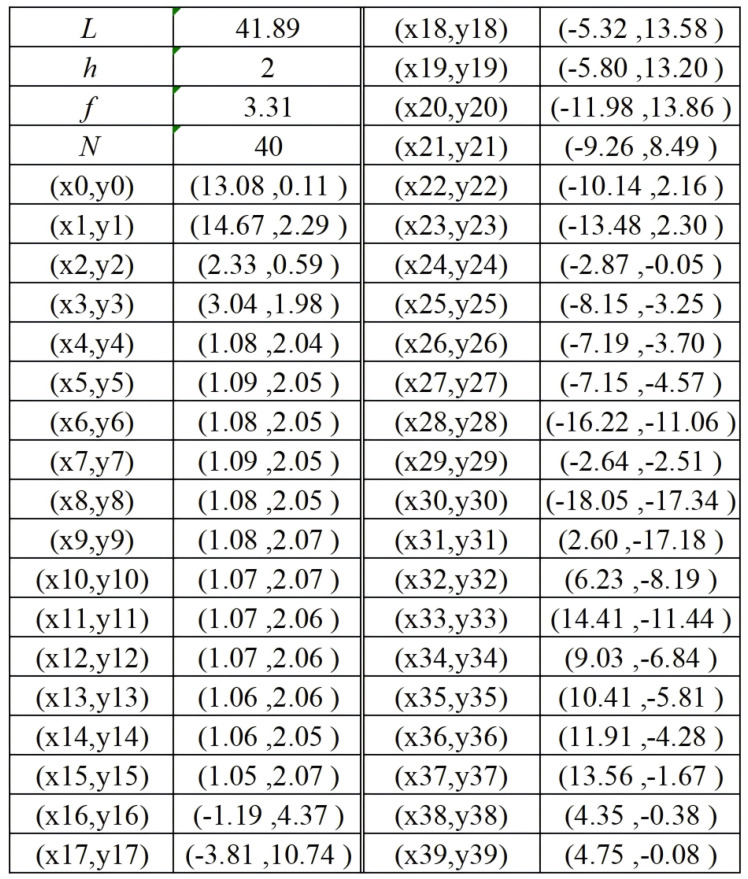
Antenna parameters.

Figure 6 presents the schematic diagram of the antenna design that achieves the widest bandwidth among all the configurations evaluated. Figure 7 provides a comprehensive listing of the essential antenna parameters, including length (*L*), height (*h*), and operational frequency (*f*). Electromagnetic simulations were performed using ANSYS Electronics Suite 2021 R2, which enabled the accurate analysis and validation of the antenna’s performance under various operating conditions.

**Figure 8:**
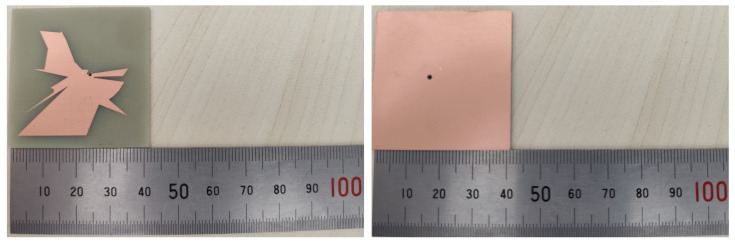
Photographs of the fabricated antenna prototype.

To verify the simulated performance of the proposed antenna, a physical prototype was fabricated. Figure 8 presents three photographs of the fabricated sample, illustrating its overall structure, key components, and detailed features. The manufacturing process closely adhered to the design specifications, ensuring that the prototype accurately represented the simulated model.

### 4.3. Antenna Design Performance Analysis

**Figure 9:**
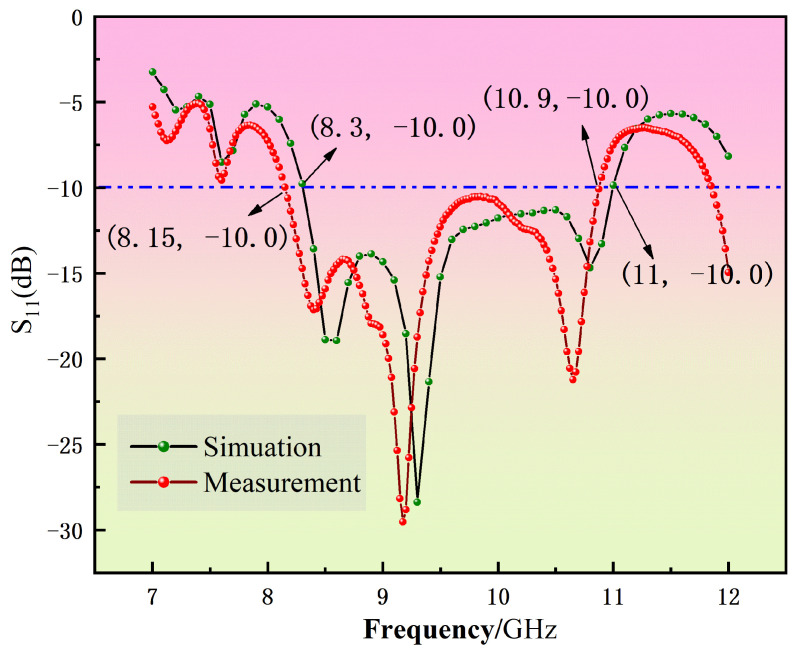
S-parametercurve of antenna obtained by simulation.

**Figure 10:**
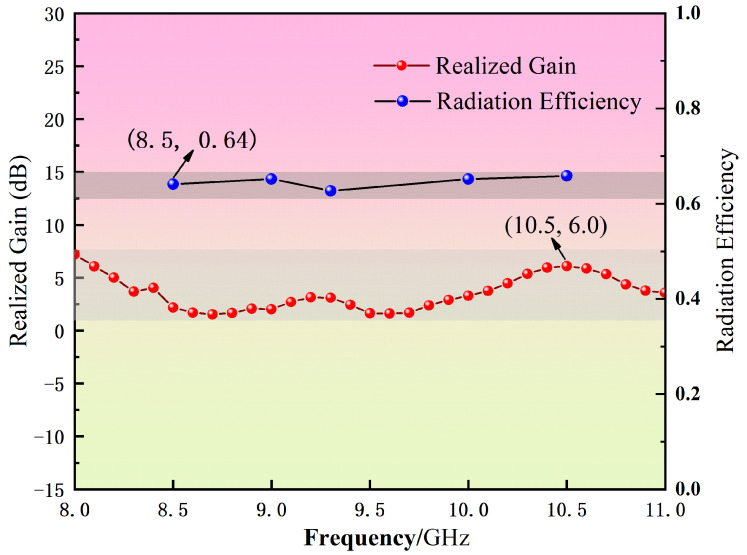
Gain and efficiency of the irregular polygon patch antenna.

Figure 9 presents both the simulated and measured S_11_ parameters of the antenna, illustrating consistent trends between simulation and experimental results. The blue dotted line is the −10 db line. The simulated operating band spans from 8.3 GHz to 11 GHz with an absolute bandwidth of 2.7 GHz, while the measured operating band spans from 8.15 GHz to 10.9 GHz with an absolute bandwidth of 2.75 GHz. The relative bandwidths are 28% and 28.9%, respectively. This validation demonstrates the efficacy of the proposed optimization algorithm in designing antennas with enhanced performance metrics suitable for applications requiring broad frequency coverage. Figure 10 illustrates the simulated variations in gain and radiation efficiency of the antenna across the specified frequency range. The gain exhibits a consistent trend within the operational bandwidth, reaching a peak value of 6 dB at 10.5 GHz. Concurrently, the radiation efficiency maintains a high performance, averaging approximately 0.64 throughout the frequency band.

**Figure 11:**
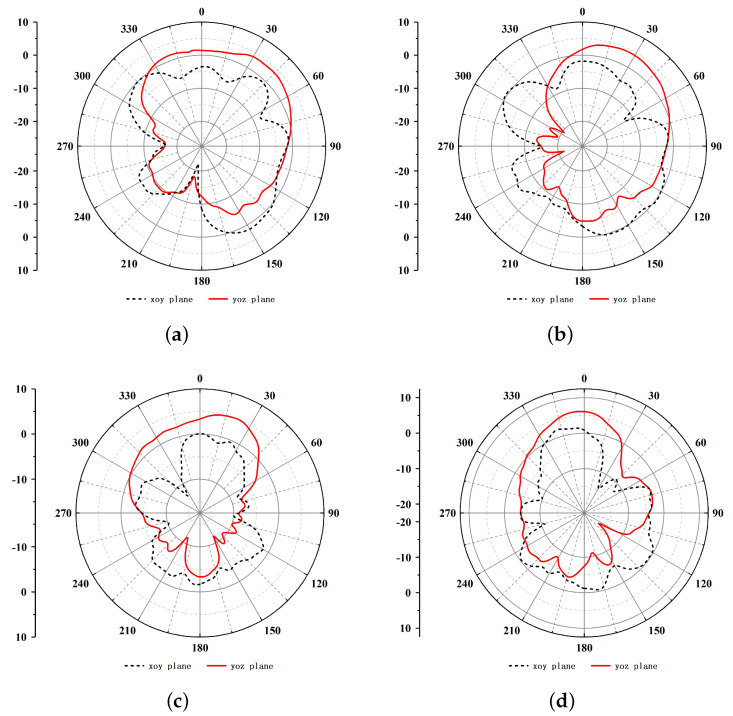
Simulated radiation patterns of the irregular polygon patch antenna. (**a**) Radiation patterns of 8.5 GHz. (**b**) Radiation patterns of 9 GHz. (**c**) Radiation patterns of 10 GHz. (**d**) Radiation patterns of 10.5 GHz.

Figure 11 presents the simulated radiation patterns for both the E-plane and H-plane of the irregular polygon patch antenna at 8.5 GHz, 9 GHz, 10 GHz and 10.5 GHz. The simulation results corroborate the efficacy of the antenna design and demonstrate the capability of the proposed intelligent optimization methodology to address complex multi-objective optimization challenges in antenna development. The implementation of adaptive variation and crossover mechanisms supports a balanced exploration and exploitation strategy, thereby facilitating the creation of compact and efficient antenna configurations with extensive operational bandwidths. bandwidths.

Table 2 provides a performance comparison between the proposed IPPA and several previously reported designs. The proposed antenna demonstrates a relative bandwidth of 27.9%, a substantial enhancement when compared to the 5.5% to 12.7% bandwidth range of the reference antennas. Furthermore, the proposed antenna achieves a superior return loss of −30 dB, which indicates more effective impedance matching than the reference designs, whose return loss values are between −17.14 dB and −23.8 dB. While the physical dimensions of the antenna are slightly larger than those of some designs in the literature, they are offset by its larger bandwidth. Overall, the proposed IPPA demonstrates enhanced bandwidth and return loss while validating an automated optimization framework.

## 5. Conclusions

In this paper, an improved NSGA-II algorithm was proposed for the multi-objective optimization of irregular polygon patch antennas. The proposed methodology addresses the persistent issues of premature convergence and diminishing population diversity in evolutionary optimization by incorporating adaptive control of genetic operator probabilities combined with simulated annealing-based selection mechanisms. The optimization framework, implemented through co-simulation with HFSS, generated physically realizable antenna designs while handling 83 independent geometric parameters. The optimized antenna configuration achieved a compact volume of 2807.6 mm^3^ with an operational bandwidth of 2.7 GHz in the X-band frequency range. Experimental measurements of fabricated prototypes demonstrated agreement with simulation results, validating both the optimization methodology and its practical implementation. The demonstrated performance improvements and experimental validation establish the proposed algorithm as an effective tool for complex antenna design problems. In the future, applying this methodology to optimize other intricate antenna geometries, such as multi-band or reconfigurable structures, presents an avenue for further demonstrating its versatility and impact in electromagnetic design. Prospective work will involve applying this methodology to optimize more intricate antenna geometries, such as multi-band or reconfigurable structures, to further validate its versatility and broaden its impact in electromagnetic design. A principal thrust of our future research will be enhancing the computational efficiency of the optimization process. One promising direction is the implementation of surrogate-assisted optimization. The development of computationally inexpensive surrogate models, based on techniques such as Kriging or artificial neural networks, can substantially mitigate the dependence on time-intensive full-wave electromagnetic simulations. Furthermore, we will investigate dimensionality reduction techniques. Such methods facilitate a more focused optimization within a lower-dimensional search space, which is anticipated to accelerate convergence.

## Figures and Tables

**Table 1:** Symbol description.

Symbol	Meaning
U(a,b)	Uniform distribution on (a,b)
${\left\{a_{i}\right\}}_{n}$	A serie or list which contain *n* elements for $a_{0}$ to $a_{n-1}$
F(A,B)	The set of all projections from A to B
M	The set of HFSS model
*L*	Side length of the substrate
*h*	Thickness of the substrate
$\left(x_{i},y_{i}\right)$	The *i*-th vertex of polygon
*f*	Distance from feed probe to the center of the substrate

**Table 2:** Performance comparison between the proposed antenna and other designs.

Designed Antenna	Frequency Band (GHz)	Patch Type	Relative Bandwidth	Size of Antenna (mm^3^)	Return Loss (dB)
Reference [35]	9.3–10.5	Rectangular	12.1%	40.0×30.0×1.6	−19.8
Reference [36]	9.2–9.8	Rectangular	6.3%	18.7×16.0×1.6	−23.8
	8.7–9.2	Triangular	5.5%	18.7×16.0×1.6	−17.8
	9.0–9.7	Circular	7.4%	18.7×16.0×1.6	−18.5
Reference [37]	10.3–11.7	Circular	12.7%	40.0×40.0×1.6	−17.1
Proposed antenna	8.3–11.0	IPPA	27.9%	41.9×41.9×1.6	−30.0

## Data Availability

The data presented in this study are available on request from the corresponding author. The data are not publicly available due to privacy.

## References

[1] Sundarsingh E.F., Velan S., Kanagasabai M., Sarma A.K., Raviteja C., Alsath M.G.N. (2014). Polygon-Shaped Slotted Dual-Band Antenna for Wearable Applications. IEEE Antennas Wirel. Propag. Lett..

[2] Saxena P., Kothari A. (2017). Mathematical Modeling of n-Sided Polygon Metamaterial Split Ring Resonators for 5.8 GHz ISM Band Applications. Wirel. Pers. Commun..

[3] Bose S., Ramaraj M., Raghavan S., Kumar S. (2012). Mathematical Modeling, Equivalent Circuit Analysis and Genetic Algorithm Optimization of an N-sided Regular Polygon Split Ring Resonator (NRPSRR). Procedia Technol..

[4] Sorokosz L., Zieniutycz W. (2012). On the Approximation of the UWB Dipole Elliptical Arms with Stepped-Edge Polygon. IEEE Antennas Wirel. Propag. Lett..

[5] Joshi A., Singhal R. Gain improvement in polygonal patch antennas. Proceedings of the 2015 Annual IEEE India Conference (INDICON).

[6] Herscovici N., Osorio M., Peixeiro C. (2002). Miniaturization of rectangular microstrip patches using genetic algorithms. IEEE Antennas Wirel. Propag. Lett..

[7] Jayasinghe J.W., Anguera J., Uduwawala D.N. (2013). A high-directivity microstrip patch antenna design by using genetic algorithm optimization. Prog. Electromagn. Res. C.

[8] Nguyen T.H., Morishita H., Koyanagi Y., Izui K., Nishiwaki S. (2014). A Multi-Level Optimization Method Using PSO for the Optimal Design of an L-Shaped Folded Monopole Antenna Array. IEEE Trans. Antennas Propag..

[9] Bianchi D., Genovesi S., Monorchio A. (2012). Constrained Pareto Optimization of Wide Band and Steerable Concentric Ring Arrays. IEEE Trans. Antennas Propag..

[10] Goudos S.K., Sahalos J.N. (2010). Pareto Optimal Microwave Filter Design Using Multiobjective Differential Evolution. IEEE Trans. Antennas Propag..

[11] Yuan Y., Chan C., Man K., Mittra R. A genetic algorithm approach to FSS filter design. Proceedings of the IEEE Antennas and Propagation Society International Symposium, 2001 Digest, Held in Conjunction with: USNC/URSI National Radio Science Meeting (Cat. No.01CH37229).

[12] Venkatarayalu N., Ray T., Gan Y.B. (2005). Multilayer dielectric filter design using a multiobjective evolutionary algorithm. IEEE Trans. Antennas Propag..

[13] Singh P., Rossi M., Couckuyt I., Deschrijver D., Rogier H., Dhaene T. (2017). Constrained multi-objective antenna design optimization using surrogates. Int. J. Numer. Model. Electron. Netw. Devices Fields.

[14] Bhattacharya S., Chattopadhyay S., Talukder S., Bag S., Mishra S., Gangopadhyaya M. Optimization of inset-fed microstrip patch antenna using genetic algorithm. Proceedings of the 2015 International Conference and Workshop on Computing and Communication (IEMCON).

[15] Dierck A., Declercq F., Vervust T., Rogier H. (2015). Design of a Circularly Polarized Galileo E6-Band Textile Antenna by Dedicated Multiobjective Constrained Pareto Optimization. Int. J. Antennas Propag..

[16] Koziel S., Ogurtsov S. (2013). Multi-Objective Design of Antennas Using Variable-Fidelity Simulations and Surrogate Models. IEEE Trans. Antennas Propag..

[17] Liu S., Wang Q., Gao R. (2014). A topology optimization method for design of small GPR antennas. Struct. Multidiscip. Optim..

[18] Wang Q., Gao R., Liu S. (2017). A novel parameterization method for the topology optimization of metallic antenna design. Acta Mech. Sin..

[19] Dutta S., Das K.N. (2019). A Survey on Pareto-Based EAs to Solve Multi-objective Optimization Problems. Soft Computing for Problem Solving.

[20] Falcón-Cardona J.G., Coello C.A.C. (2020). Indicator-based Multi-objective Evolutionary Algorithms: A Comprehensive Survey. ACM Comput. Surv..

[21] Trivedi A., Srinivasan D., Sanyal K., Ghosh A. (2017). A Survey of Multiobjective Evolutionary Algorithms Based on Decomposition. IEEE Trans. Evol. Comput..

[22] Shirajuddin T.M., Muhammad N.S., Abdullah J. (2023). Optimization problems in water distribution systems using Non-dominated Sorting Genetic Algorithm II: An overview. Ain Shams Eng. J..

[23] Wang X., Zhu H., Luo X., Chang S., Guan X. (2024). A novel optimal dispatch strategy for hybrid energy ship power system based on the NSGA-II. Electr. Power Syst. Res..

[24] Arani M.S., Shahidi R., Zhang L. (2024). A State-of-the-Art Survey on Advanced Electromagnetic Design: A Machine-Learning Perspective. IEEE Open J. Antennas Propag..

[25] Pietrenko-Dabrowska A., Koziel S. (2023). Dimensionality-reduced antenna modeling with stochastically established constrained domain. Knowl. Based Syst..

[26] Xie J., Chen S., Cai J., Zhao P., Cheng Y., Liu J., Wang G. A Wideband GaAs Double-balanced Mixer using Genetic Algorithm based Optimization. Proceedings of the 2023 16th UK-Europe-China Workshop on Millimetre Waves and Terahertz Technologies (UCMMT).

[27] Cheng Y.F., Shao W., Ding X., Yu M., Ma J., Jin C. (2017). A novel beam steerable antenna based on dual-reconfiguration technique. J. Electromagn. Waves Appl..

[28] Li Y., Yang S., Ren Z. (2024). A Multi-Objective Topology Optimization Methodology Using Deep Learning and Its Application to Electromagnetic Devices. IEEE Trans. Magn..

[29] Huang J., Li W., He Y., Zhang L., Wong S.W. Optimization of Antenna Design Using the Artificial Neural Network and the Simulated Annealing Algorithm. Proceedings of the 2021 Computing, Communications and IoT Applications (ComComAp).

[30] He Y., Huang J., Li W., Zhang L., Wong S.W., Chen Z.N. (2024). Hybrid Method of Artificial Neural Network and Simulated Annealing Algorithm for Optimizing Wideband Patch Antennas. IEEE Trans. Antennas Propag..

[31] Mishra B., Verma R.K., Yashwanth N., Singh R.K. (2022). A review on microstrip patch antenna parameters of different geometry and bandwidth enhancement techniques. Int. J. Microw. Wirel. Technol..

[32] Alnahwi F.M., Al-Yasir Y.I.A., Ali N.T., Gharbia I., See C.H., Abd-Alhameed R.A. (2022). A Compact Wideband Circularly Polarized Planar Monopole Antenna With Axial Ratio Bandwidth Entirely Encompassing the Antenna Bandwidth. IEEE Access.

[33] Mahendran K., Gayathri D., Sudarsan H. (2021). Design of multi band triangular microstrip patch antenna with triangular split ring resonator for S band, C band and X band applications. Microprocess. Microsyst..

[34] Deb K., Pratap A., Agarwal S., Meyarivan T. (2002). A fast and elitist multiobjective genetic algorithm: NSGA-II. IEEE Trans. Evol. Comput. TEC.

[35] Borazjani O., Naser-Moghadasi M., Rashed-Mohassel J., Sadeghzadeh R.A. (2020). Bandwidth improvement of planar antennas using a single-layer metamaterial substrate for X-band application. Int. J. Microw. Wirel. Technol..

[36] Kiruthika R., Shanmuganantham T. Comparison of different shapes in microstrip patch antenna for X-band applications. Proceedings of the 2016 International Conference on Emerging Technological Trends (ICETT).

[37] Islam M.M., Islam M.T., Faruque M.R.I., Hueyshin W. Design of an X-band microstrip patch antenna with enhanced bandwidth. Proceedings of the 2013 2nd International Conference on Advances in Electrical Engineering (ICAEE).

